# Late recurrence of Wilms tumor after a 32−year disease−free interval: case report and literature review

**DOI:** 10.3389/fonc.2026.1803421

**Published:** 2026-04-10

**Authors:** Matthew R. Kroll, Rachaita Lakra, Jonathan J. Davick, Jenna Gedminas, Mohammed Milhem, Sara C. Horton

**Affiliations:** 1 Department of Hematology, Oncology, and Bone Marrow Transplant, University of Iowa Hospitals and Clinics, Iowa, IA, United States; 2 Stead Family Children’s Hospital, Carver College of Medicine, University of Iowa Hospitals and Clinics, Iowa, IA, United States; 3 Division of Pediatric Hematology and Oncology, Department of Pediatrics, University of Iowa Hospitals and Clinics, Iowa, IA, United States

**Keywords:** CTNNB1, late recurrence, nephrogenic rests, pediatric renal malignancy, TP53, Wilms tumor

## Abstract

**Background:**

Wilms tumor (WT) is the most common pediatric renal malignancy, but carries a significantly worse prognosis in the adult population. Late recurrence (LR) of a primary WT, defined as disease recurrence five or more years after initial diagnosis, is rare and does not have standardized treatment regimens outside the pediatric population. In this paper we report a LR 32 years after diagnosis, the longest documented disease-free interval ever described.

**Case presentation:**

A one-year-old female patient was diagnosed with bilateral, favorable-histology WT and treated with chemotherapy. Biopsies of residual masses after treatment confirmed benign tissue, and she was observed with serial imaging for seventeen years without evidence of recurrence. Thirty-two years after initial diagnosis, she presented with gross hematuria and right-sided abdominal pain. Biopsy demonstrated of the left renal mass was consistent with a LR of WT. She received neoadjuvant chemotherapy followed by left partial nephrectomy, right radical nephrectomy, and adjuvant radiation therapy and remains cancer free without any evidence of tumor recurrence.

**Conclusion:**

This case of a 32-year treatment-free interval prior to LR is the longest such case in the medical literature. This case highlights the importance of multidisciplinary management in this rare clinical scenario of LR WT.

## Introduction

Wilms tumor (WT) is the most common pediatric renal malignancy with a median age at diagnosis of 3.5 years (1). Adult WT is rare, accounting for less than 1% of adult renal neoplasms (2). Pediatric and adult WTs are histomorphologically and radiographically identical, yet a significant overall survival difference exists between the two groups, 92% versus 68% respectively (3–5). This outcome gap may be driven by the unfamiliarity of adult oncologists and pathologists with WT, causing delayed diagnosis and intervention (6). Some of these adult patients present as late recurrences (LR) of pediatric tumors. A late recurrence, defined as a tumor recurrence 5 years after diagnosis, is exceedingly rare, estimated at a rate of 0.5% of all cases of all recurrences. with a median time of relapse of 13.2 years. Previously, the longest recorded disease-free interval for a LR 25 years (7, 8). We present an unusual case of LR of a bilateral WT thirty-two years after initial diagnosis.

## Case description and diagnostic assessment

A one-year-old female was diagnosed with biopsy-confirmed, localized, bilateral, favorable-histology WT. She was treated on clinical trial CCG-461 with doxorubicin, dactinomycin and vincristine (DD-4A). Due to the presence of residual, unresectable disease, chemotherapy was intensified to ifosfamide and etoposide, followed by carboplatin and bleomycin. Repeat biopsies of the residual mass demonstrated benign tissue. She completed chemotherapy with DD-4A. No surgical resection of or radiation to the residual masses was performed. She was observed with serial imaging for seventeen years after the end of her therapy. No changes in the sizes of the residual masses were reported during this interval.

**Figure 1:**
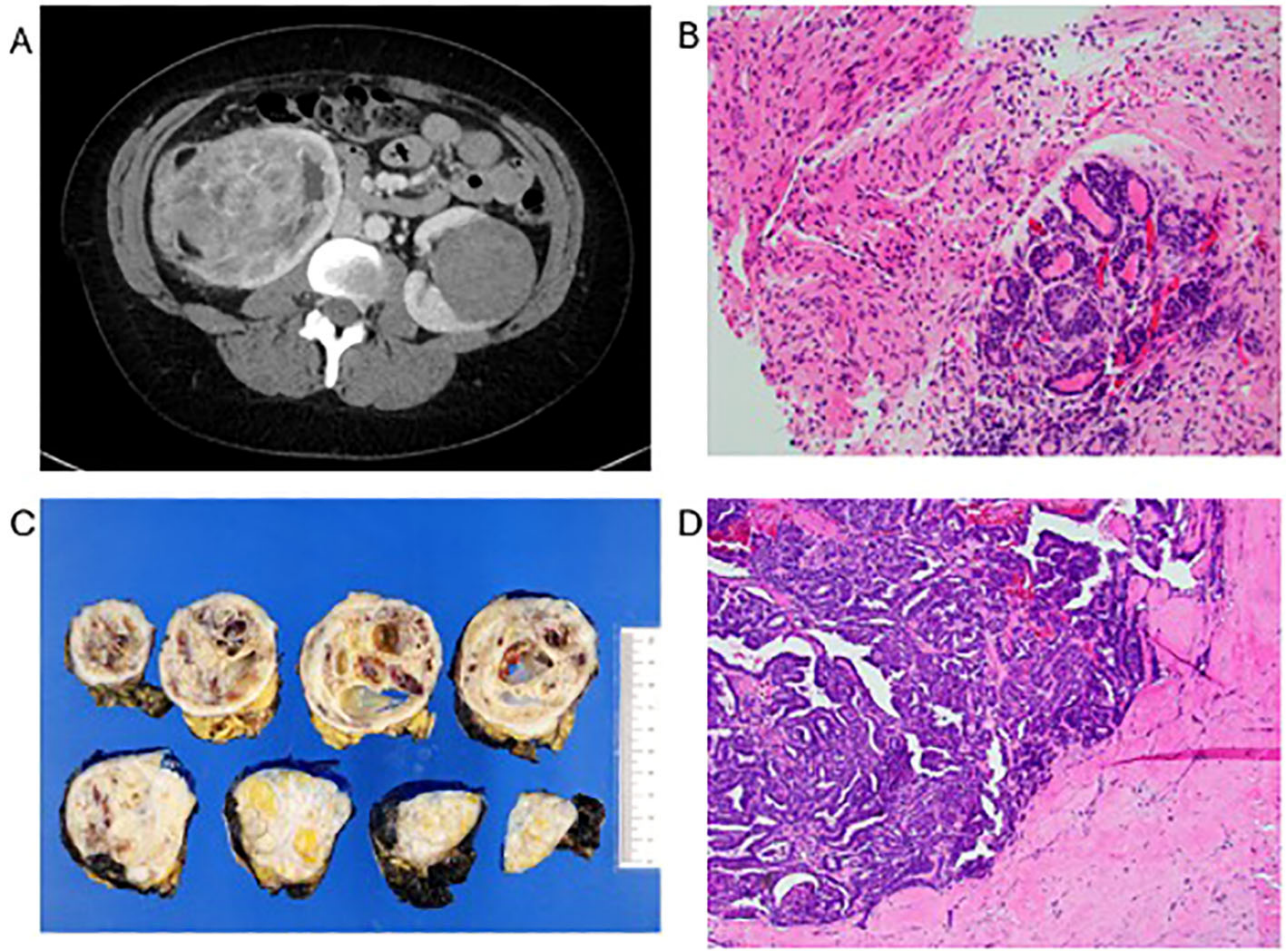
**(A)** Axial CT imaging showing bilateral renal masses. **(B)** Biopsy of right kidney demonstrating epithelial and stromal elements. **(C)** Right kidney post-treatment resection gross cut surfaces. **(D)** Right kidney post-treatment resection specimen showing residual tubules and treatment effect.

Thirty-two years after her initial diagnosis, she presented to a local hospital following an episode of painless gross hematuria and right sided abdominal pain. Physical examination demonstrated a palpable mass in the lower-right quadrant of her abdomen. Subsequent CT scan of her abdomen and pelvis demonstrated an increase in size of her previously stable bilateral renal masses. The right lower-pole mass, previously measuring 4.2 x 3.8 cm 15 years prior, measured 9.3 cm x 8.2 cm x 10.6 cm. The left mid-pole mass, previously 3.8 cm x 3.5 cm 15 years prior, was now 6.5 cm x 6.4 cm. A smaller renal mass on the left lower-pole also grew within this timespan, from 1.6 cm x 1.5 cm to 2.3 cm x 1.9 cm (**Figure 1A**). Core needle biopsy of the right renal mass (**Figure 1B**) demonstrated tumor cells with strong PAX8 expression and lacking WT-1 on immunohistochemistry, consistent with a LR of WT. Massively parallel next generation DNA sequencing revealed the presence of variant CTNNB1, SMC1A genes, and a pathologic TP53 c.313G>A mutation. There was no evidence of anaplasia. The tumor was deemed to have favorable histology, with the caveat that the biopsy may not represent histology of the entire mass.

Neoadjuvant chemotherapy was initiated using DD-4A. Following 4 cycles of DD-4A, repeat CT scan of the chest abdomen and pelvis showed a decrease in size of the right renal mass from 9.3 cm x 8.2 cm to 7.7 cm x 6.8 cm, the left mid-pole mass from 6.5 cm x 6.4 cm to 5.1 cm x 4.4 cm, and the left lower-pole mass from 2.3 x 1.9 cm to 2.0 cm x 1.6 cm. She then underwent a left partial nephrectomy with final pathology consistent with favorable histology WT with teratoid features. The tumor showed predominantly epithelial and stromal differentiation with no blastemal cells, and demonstrated no definite necrosis, consistent with an SIOP intermediate risk posttherapy histology. Two months later she underwent a right radical nephrectomy. Histopathology of the newly resected right renal mass was similar to the previously resected left renal mass and with SIOP intermediate risk posttherapy histology (**Figures 1C, D**). Repeat CT scan demonstrated stable post-surgical changes without any evidence of recurrent tumor.

Despite indication for adjuvant chemotherapy, further systemic treatment was deferred due to the risk of developing chemotherapy related toxicities. Adjuvant radiation of 10.8 Gy in 6 fx to the right flank was performed as the right renal mass was biopsied and removed in a piecemeal excision, warranting a COG local stage III (9).

**Figure 2:**
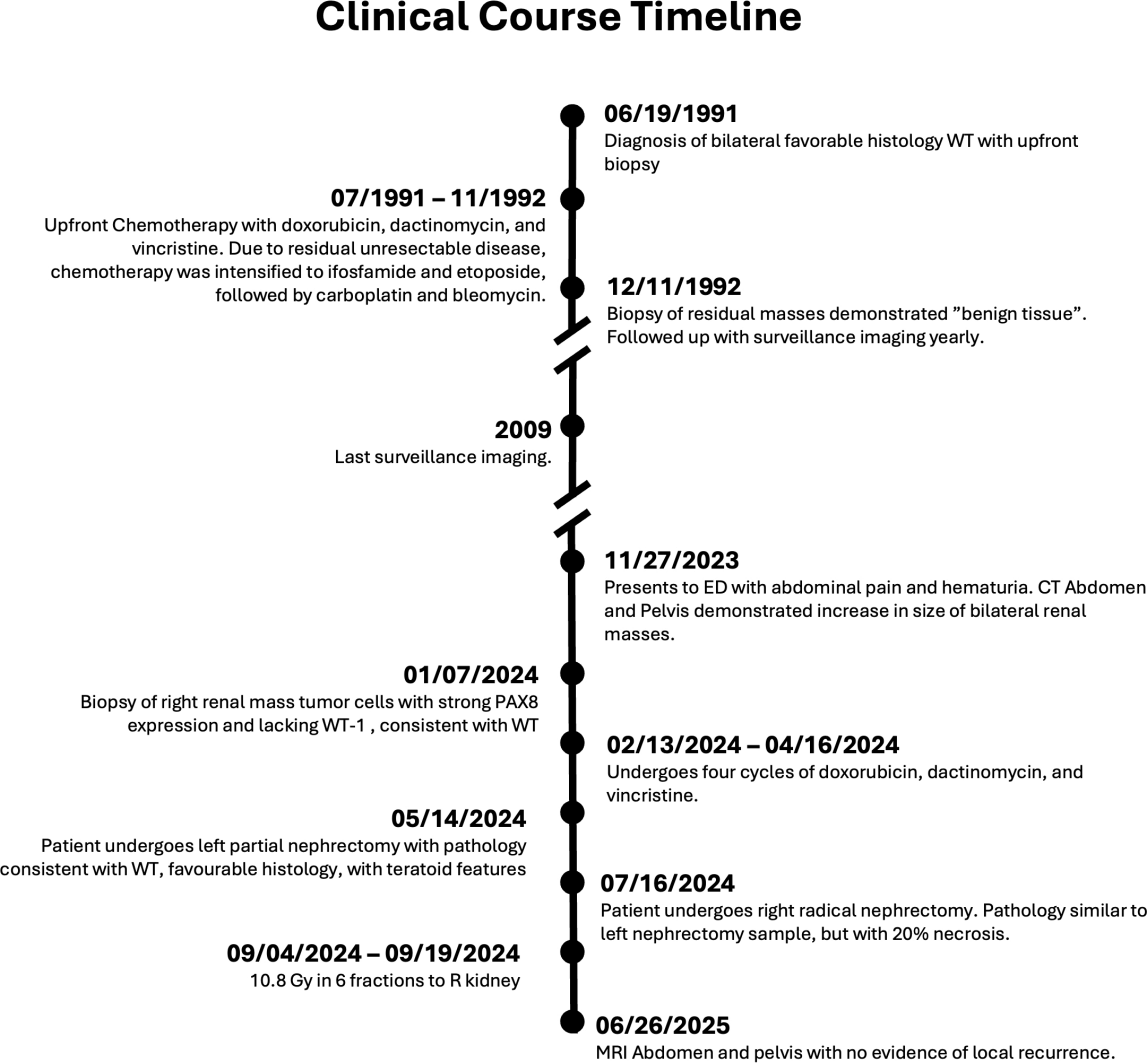
Clinical course of patient.

One year after confirmed relapse, imaging showed stable postsurgical changes without any evidence of local tumor recurrence. The clinical course is summarized in timeline form in **Figure 2**.

## Discussion

As medical advances have pushed the life expectancy of patients diagnosed with WT beyond five years, more cases of LR will likely be reported. Malogolowkin et al. retrospectively analyzed ten clinical trials to characterize LRs. Their group found that only 70 out of 13,330 WT patients (0.5%) had a confirmed LR with a median time to recurrence of 13.2 years from initial diagnosis. The clinical outcomes of LRs are highly variable, with estimates extrapolated from single center case series and case reports; however, when a LR occurs in the contralateral kidney, outcomes are better. One study demonstrated a similar overall survival to primary WT (87% cure rate in contralateral kidney LR vs 45% in other sites of LR) (7).

LRs are divided into true LR, which arise from the surviving cells of a primary WT, and metachronous primary WTs, which are due to the persistence of clusters of embryonal cells, called nephrogenic rests (NR) not effected by previous treatment. NRs persist abnormally after renal development and are found in approximately 1% of infant kidneys at autopsy (10). When compared to true recurrences, metachronous disease tends to happen later, with a median time to recurrence of 13.0 months to 23.1 months respectively. 96.2% of metachronous recurrences happen within the first 5 years from diagnosis (11). However, there are cases in the literature of metachronous disease diagnosed 13.1 years from the initial diagnosis (12). The location of NRs on the kidney can impart unique characteristics to the NRs. Perilobar nephrogenic rests (PLNR) are associated with bilateral synchronous disease, and intralobar nephrogenic rests (ILNR) are associated with metachronous disease. In patients presenting with bilateral synchronous disease, such as the patient discussed above, 74-79% have evidence of PLNR on biopsy. In comparison, only 34 – 41% of these patients have evidence of ILNR. Conversely, in bilateral metachronous disease, 42% of biopsies have evidence of PLNR and 63 – 75% have evidence of ILNR (13). There is no data in the literature about the prevalence of NR in the adult population, but it is theorized that most LR cases may arise from persistent NR. In our discussed case, we cannot differentiate if this tumor stems from previously dormant and pretreated malignant cells, or persistent NRs. However, as the masses seem to arise from previously seen masses, it is likely this case represents a true LR.

Most incidences of LR occur during puberty, possibly due to hormonal changes activating quiescent WT cells. The evidence base for this mechanism of relapse is small, however a previously published case demonstrated estrogen and progesterone receptor positivity on IHC of a LR WT resected from an 11-year-old female (7). In our case, 32 years after initial diagnosis, no clear trigger was identified that activated the growth in previously quiescent cells.

A seminal investigation by Zuppan et al. characterized the pre- and post-treatment histology of WT. His team noted that after therapy, while the less mature, blastic cells of the WT tend to be eradicated with chemotherapy, more resistant, mature cancerous cells tend to be less effected and persist (14). Zuppan’s observations are consistent with what is seen in the preceding case. The initial tumor that the patient had at age 1 was a favorable histology WT without teratoid features, but her adult pathology demonstrated teratoid features. Furthermore, when her recurrent tumor was resected, there was only very mild treatment effect. Taken together, the previously unseen teratoid features and the minimal treatment effect suggest that her tumor may have come from mature, less chemo-sensitive elements of her original pediatric tumor.

The mutation profile, of this patient’s tumor, specifically the TP53 and CTNNB1 mutations, offer insight into the tumor’s behavior. TP53 mutations are strongly associated with diffuse anaplastic WT and are rarely in favorable histology tumors, like the one reported above. TP53 abnormalities confer an increased risk of recurrence and death compared to TP53-wild-type tumors. Interestingly, when TP53 mutations are present in non-anaplastic WT, the mutation may represent a progression event linked to the development of anaplasia (15). CTNNB1 mutations are found in approximately 15% of WT (16). CTNNB1 mutations activate the WNT/β-catenin signaling pathway. Tumors harboring these mutations, such as the tumor discussed in the case above, demonstrate poor volumetric response to preoperative chemotherapy (17). While neither TP53 nor CTNNB1 are clinically actionable at this time, they both provide insight into the behavior of the discussed patient’s LR.

Due to the rarity of LR of WT, no guidelines exist, and therapy is guided by expert opinion. Treatment modalities for adult WT are adopted from pediatric regimes formulated via the International Society of Pediatric Oncology (SIOP) and the Children’s Oncology Group (COG) including surgical resection through radical nephrectomy, radiotherapy and systemic therapies; however, many of these regimens have worse reported outcomes in the adult population (5). For example, in the SIOP 93–01 study, of the 30 adult WT patients, EFS was only 57% and OS was only 83% (median follow up 4 years) (18). Many different approaches to treating adult WT and LR of WT are reported in the literature (**Table 1**). Improved outcomes are reported in recurrent WT with favorable histology, lower disease stage, lack of prior radiotherapy, and initial chemotherapy with actinomycin-D and vincristine (19). Due to the limited data sets, many questions about LRs remain unanswered, such as the discrepancy between latency periods in tumors with similar stage and histology. Furthermore, there are no readily available methods for discerning LR from a contralateral metachronous primary WT. Further studies need to be performed to understand the difference in these two groups and help formalize treatment paradigms. Another area of interest is identifying similarities between cases of LR, which could help us select patients for increased surveillance past the standard five years.

**Table 1:** Published treatment regimens for adult WT and LR WT.

Case reports	Gallego-Melcón et al (19)	Radhakrishnan et al (20)	Gottesman et al. (21)	Senetta et al (22)	Lee et al (8)	Our patient
Age at initial diagnosis	6	4	5	10 months	3	1
Sex	M	F	M	F	F	F
Stage	I	I	Unknown	III	III	V
Histology at initial diagnosis	Classic Wilms tumor with no evidence of anaplasia	Classic Wilms tumor with no evidence of anaplasia	Predominant blastematous with tubular elements	Rhabdomyoblastic differentiation	Predominantly tubular epithelial component	Favorable histology Wilms tumor
Treatment	Sx + CT + RT	Sx + CT	Sx + RT	Sx + CT	Sx + CT	CT
Time to relapse from initial diagnosis (yrs)	20	20	23	23	25	32
Relapse Site	Lungs	Retroperitoneum	Tumor bed (LUQ), peritoneal carcinomatosis	Retroperitoneum	Pelvic cavity	Bilateral Kidneys
Relapse treatment	Sx + CT (type unknown)	Sx + CT (ICE) + RT	CT (VCR, ACTI -D ADR)	Sx	Sx + CT (NWTS-5) + RT + Autologous SCT	Sx + CT (DD-4A)
Outcome	Unknown	Disease free at 2 years of follow up	Died of progressive disease in 2 months	Disease free at 9 months of follow up	Disease free at 14 months of follow up	Disease free at 1 year follow up

ACTI-D, Actinomycin D; ADR, Adriamycin; CT, Chemotherapy; ICE, Ifosfamide /Carboplatin/Etoposide; LUQ, Left upper quadrant; RT, Radiotherapy; SCT, Stem Cell Transplant; Sx, Surgery; VCR, Vincristine. DD-4A, doxorubicin, dactinomycin and vincristine.

## Conclusion

This report describes the most prolonged treatment−free interval preceding a late recurrence of Wilms tumor documented to date, occurring thirty−two years after initial diagnosis. Because late recurrence remains exceptionally rare and is primarily reported through isolated cases and small institutional series, there is limited guidance to inform clinical decision−making in this setting. Our case underscores the ongoing uncertainties surrounding the optimal management of adult Wilms tumor recurrence, including the role of multimodal therapy and the prognostic implications of tumor genetics.

The marked divergence in histologic features between the patient’s childhood tumor and her adult recurrence alongside the presence of TP53 and CTNNB1 mutation highlights the biological complexity of long−latency disease and suggests that dormant or treatment−resistant cellular subpopulations may contribute to very late relapse. Broader genomic comparisons between primary tumors and their late recurrences are needed to clarify mechanisms of latency, therapeutic resistance, and progression.

This case further emphasizes the necessity of reconsidering long−term surveillance strategies for Wilms tumor survivors, particularly those with bilateral disease or persistent nephrogenic rests. As survival improves and more patients reach adulthood decades after initial treatment, standardized guidelines for extended follow−up will become increasingly important. Ultimately, accumulating and integrating cases such as this will be essential to refining risk stratification, counselling survivors, and developing evidence−based approaches to this rare but clinically significant phenomenon.

## Data Availability

The original contributions presented in the study are included in the article/**Supplementary Material**, further inquiries can be directed to the corresponding author/s.
